# A Deep Learning Approach for MIMO-NOMA Downlink Signal Detection

**DOI:** 10.3390/s19112526

**Published:** 2019-06-02

**Authors:** Chuan Lin, Qing Chang, Xianxu Li

**Affiliations:** 1 School of Electronic and Information Engineering, Beihang University, Beijing 100191, China; lclkzjp@hotmail.com; 2 State Grid Information & Telecommunication Branch, Beijing 100761, China; lixianxu@buaa.edu.cn

**Keywords:** 5G, non-orthogonal multiple access (NOMA), multiple-input multiple-output (MIMO), deep learning (DL), wireless communication

## Abstract

As a key candidate technique for fifth-generation (5G) mobile communication systems, non-orthogonal multiple access (NOMA) has attracted considerable attention in the field of wireless communication. Successive interference cancellation (SIC) is the main NOMA detection method applied at receivers for both uplink and downlink NOMA transmissions. However, SIC is limited by the receiver complex and error propagation problems. Toward this end, we explore a high-performance, high-efficiency tool—deep learning (DL). In this paper, we propose a learning method that automatically analyzes the channel state information (CSI) of the communication system and detects the original transmit sequences. In contrast to existing SIC schemes, which must search for the optimal order of the channel gain and remove the signal with higher power allocation factor while detecting a signal with a lower power allocation factor, the proposed deep learning method can combine the channel estimation process with recovery of the desired signal suffering from channel distortion and multiuser signal superposition. Extensive performance simulations were conducted for the proposed MIMO-NOMA-DL system, and the results were compared with those of the conventional SIC method. According to our simulation results, the deep learning method can successfully address channel impairment and achieve good detection performance. In contrast to implementing well-designed detection algorithms, MIMO-NOMA-DL searches for the optimal solution via a neural network (NN). Consequently, deep learning is a powerful and effective tool for NOMA signal detection.

## 1. Introduction

Since the concept of non-orthogonal multiple access (NOMA) transmission was proposed, there have been at least three categories of non-orthogonal multiple access: power-domain NOMA, code-domain NOMA, and hybrid-domain NOMA. In this article, we focus on power-domain NOMA for downlink multiuser multiple-input multiple-output (MIMO) systems on account of its representativeness and typicality of the non-orthogonal multiple access technique. Results in this paper can offer a reference for deep learning on other more complicated NOMA techniques. Meanwhile, with the benefit of the MIMO technique, MIMO-NOMA can enhance the throughput of the communication system [1].

To date, the most universal method for MIMO-NOMA signal detection has been successive interference cancellation (SIC) reception. In the downlink, a multiuser signal is first multiplexed by superposition coding with a total power constraint at the base station. At the receiver, a signal suffering from channel impairment will invoke the SIC mechanism in order to decode the expected signal depending on the order of the user equipment (UE) channel gains in a cluster. The UE signal with poorer channel gain is allocated higher transmit power and is decoded first, while the UE signal with lower power allocation is treated as interference. After the signal with higher power is detected and decoded correctly, a modulation reconstruction is executed. Then, the reconstructed signal is subtracted from the received signal. The process continues until the UE can decode its desired information. From an information-theoretic perspective, SIC is an optimal multiple-access scheme in terms of the achievable multiuser capacity region in both uplink and downlink [2].

Instead of the classic SIC method, we resort to the power of deep learning—multilayer neural networks (NNs)—to achieve MIMO-NOMA signal detection. As one branch of machine learning, deep learning has made substantial progress in recent years and has been applied to many fields. The major evolution of deep learning mainly involves deep neural networks (DNNs) in pattern classification and recognition, conventional neutral networks (CNNs) in image processing, and recurrent neural networks (RNNs) in voice recognition for natural language processing (NLP). The powerful performance of deep learning has considerably changed our daily lives and the cognition of artificial intelligence (AI).

Note that although Graphic Processing Unit (GPU) resources are still somewhat expensive for users, the cost in recent years has been in steady decline according to Moore’s law. New products with higher performance and lower cost continue to spring up. Tensor processing units (TPUs), a customized ASIC chip for AI, were proposed by Google in 2016. It is reported that the cost of third-generation TPUs could be lower by 80%. We firmly believe that the era of AI is coming.

In the field of wireless communication, although successful commercial applications related to deep learning are relatively less abundant [3], many researchers are attempting to introduce machine learning techniques into communication systems, especially for improving existing signal processing algorithms. In channel decoding, deep learning can learn a form of decoding algorithm rather a simple classifier, and successful examples have been reported, such as decoding linear code [4] and polar code [5]. Ref. [6] improves the standard belief propagation (BP) decoder to a BP-CNN and achieves improved bit error rate (BER) performance with low complexity. Compressed sensing (CS) based on deep learning theory can outperform the state-of-the-art CS-based methods in terms of both recovery quality and computation time [7]. Modulation classification and identification based on deep learning is also a popular area of research [8]. In the realm of mobile networking, the schematic of the stacked auto-encoder (SAE) works well in the applications of feature learning, protocol classification, and anomalous protocol detection [9]. Mobile traffic classifiers based DL have also been reported to handle encrypted traffic and reflect their complex traffic patterns [10,11]. Achievements in blind detection for MIMO systems with deep learning are also continuously reported [12,13]. Additionally, a scheme integrating DL into an orthogonal frequency division multiplexing (OFDM) system has been put forward [14], and its numerical results revealed the potential performance of DNNs. A fully connected end-to-end DL system including an encoding layer, noise layer, and decoding layer was reported in [15] for MIMO, and [16] designed a long short term memory (LSTM) network for uplink NOMA to realize the end-to-end transmission as well. Their results showed the excellent performance of the autoencoder for jointly learning transmit and receive functions. However, in most realistic scenarios, it is difficult to synthetically train and optimize both send and receive functions of the DL system.

Traditionally, the SIC method is affected by the error propagation (EP) and receiver complexity related to the number of UEs. We propose a learning method based on a DNN to efficiently estimate the channel state and decode the expected signal. Its benefits not only lie in the performance gain in the communication system, but in the reduction of reference signal overhead to increase the throughput in the downlink system.

The main contributions of this paper are summarized as follows:

1. To the best of our knowledge, we designed the first downlink MIMO-NOMA detecting system based on DL methods. The proposed system can process the traditional MIMO-NOMA signal directly instead of implementing an SIC receiver.
2. We take full advantage of the ability of DNNs to process high-dimensional data. The detection performance can be improved by the DL-based method.
3. The MIMO-NOMA-DL system can estimate the characteristics of the MIMO Rayleigh fading channel and then decode the signal. The process of channel estimation and signal detection can be considered synthetically instead of separately.
4. We evaluated the performance of the proposed system. Results comparable to traditional MIMO-NOMA based on SIC were obtained.
5. We provide relevant simulation results on the impact of some key parameters. These parameters include the modulation type, power allocation, and mini-batch. The proposed system outperformed the traditional SIC method in all testing situations.

The remainder of this paper is structured as follows: In Section 2, the necessary background on MIMO-NOMA system and DL is briefly reviewed. Then, the proposed MIMO-NOMA-DL system is depicted in detail, including the feasibility analysis, overall structure, and DNN design in Section 3, followed by numerical studies in Section 4. Finally, Section 5 concludes the paper and discusses future works.

*Notations*: Vectors are denoted by boldface small letters, while matrices are denoted by boldface capital letters. Superscripts ${\left(\cdot \right)}^{*}$, ${\left(\cdot \right)}^{T}$, ${\left(\cdot \right)}^{H}$, ${\left(\cdot \right)}^{-1}$ and ||·|| represent the conjugate, transpose, Hermitian transpose, inverse, and Frobenius norm operators, respectively. Also, $R^{N\times M\times K}$ denotes the vector space of all N×M×K real matrices and $H_{\left(n\right)}$ is the node-n matricization.

## 2. Fundamentals of MIMO-NOMA and the Deep Learning System

In this section, we describe the fundamental theory of NOMA transmission and the traditional SIC algorithm. Then, we analyze the basic architecture of deep learning. For simplicity, we assumed that the bandwidth is 1 Hz. The channel impairment includes Rayleigh fading channel and the additive white Gaussian noise (AWGN)  channel.

### 2.1. MIMO-NOMA Basics

**Figure 1:**
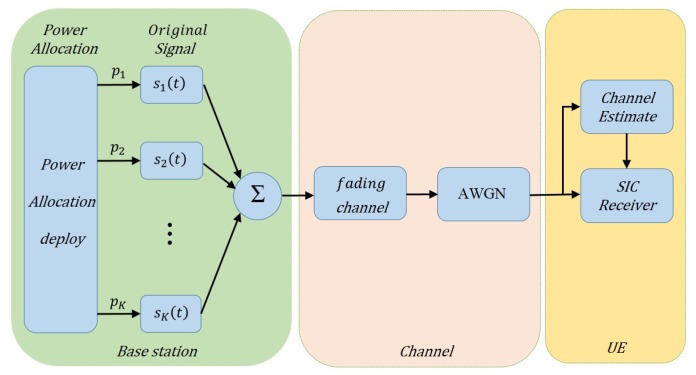
Non-orthogonal multiple access (NOMA) system architecture. AWGN: additive white Gaussian noise; SIC: successive interference cancellation.

NOMA can implement multiple-signal multiplexing by allocating unbalanced power within a same time/frequency/code resource. In contrast to traditional orthogonal multiple access (OMA) using orthogonal resources such as OFDM, NOMA utilizes power in a non-orthogonal form to substantially enhance the spectrum efficiency at the expense of receiver complexity. Figure 1 shows the overall architecture of the basic NOMA system.

**Figure 2:**
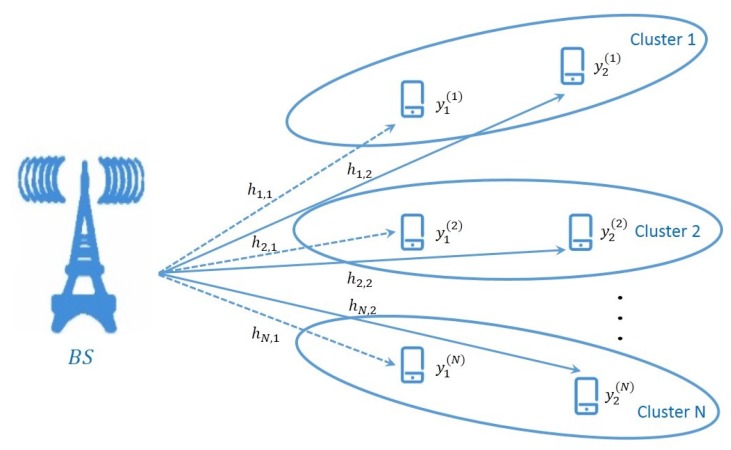
System block diagram of the multibeam multiple-input multiple-output (MIMO)-NOMA. BS: base station.

In conventional downlink multiuser MIMO, UEs generally occupy orthogonal resources. Interbeam interference can be completely eliminated when the number of base station (BS) transmit antennas is equal to or greater than the number of receive antennas [17]. In the MIMO-NOMA system, however, the total number of UE antennas is always greater than the number of transmit antennas, and these UEs have to share a cluster. Thus, interference from other UEs in the same cluster is inevitable. Multiple antennas at the base station (BS) can radiate multiple beams to form different directions via beamforming technology. A schematic is shown in Figure 2. For simplicity, in this paper, we focus on signal detection from UEs in a single cluster.

Supposing that the number of users is *K*, the *i*-th UE signal at the BS can be denoted as $s_{i}\left(t\right)$, (i=1,2,⋯,K). The power allocated to user *i* is denoted as $p_{i}$, and the transmission power is limited by the total power *P*, where $P=p_{1}+p_{2}+\cdots +p_{K}$. The total transmission signal can be expressed as:

(1) $$s\left(t\right)=\sum_{i=1}^{K}\sqrt{p_{i}}s_{i}\left(t\right).$$

During power allocation, various strategies can be followed for different situations and destinations. A detailed analysis of these strategies can be seen in [18]. The signal received through the fading channel and AWGN channel can be denoted as:

(2) $$y\left(t\right)=h\left(t\right)*\sum_{i=1}^{K}\sqrt{p_{i}}s_{i}\left(t\right)+n\left(t\right).$$

**Figure 3:**
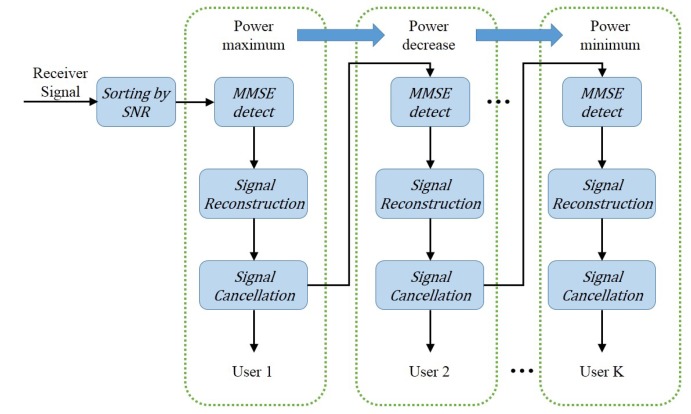
SIC receiver architecture. SNR: signal-to-noise ratio.

As mentioned previously, the detection of NOMA usually adopts the SIC method, and NOMA with a SIC has shown to be an optimal multiple-access scheme in terms of the achievable multiuser capacity region in both uplink and downlink [2]. The SIC processes are shown in Figure 3.

At the receiver, the SIC process is executed in descending order of signal-to-noise ratio (SNR). For example, for $p_{1}>p_{2}>\cdots >p_{K}$, user 1 will be decoded directly while the other signals are viewed as noise. The throughput can be expressed as:

(3) $$R_{1}=log_{2}\left(1+\frac{p_{1}{\left|h_{1}\right|}^{2}}{\sum_{i=2}^{K}p_{i}{\left|h_{1}\right|}^{2}+N_{0}}\right).$$

For user k∈[1,K], assuming that the first k−1 users are decoded perfectly, the throughput for user *k* is:

(4) $$R_{k}=log_{2}\left(1+\frac{p_{k}{\left|h_{k}\right|}^{2}}{\sum_{i=k+1}^{K}p_{i}{\left|h_{k}\right|}^{2}+N_{0}}\right).$$

Hereby, the throughput for user *K* is:

(5) $$R_{K}=log_{2}\left(1+\frac{p_{K}{\left|h_{K}\right|}^{2}}{N_{0}}\right).$$

Clearly, the decoding error of the higher-power signal is accumulated and affects the decoding accuracy of the lower-power signal. This is a key problem of the SIC method that must be solved.

### 2.2. Deep Learning Basics

The main techniques of deep learning include DNN, CNN, and RNN. In this section, the basics of these DL-based approaches are briefly introduced.

**Figure 4:**
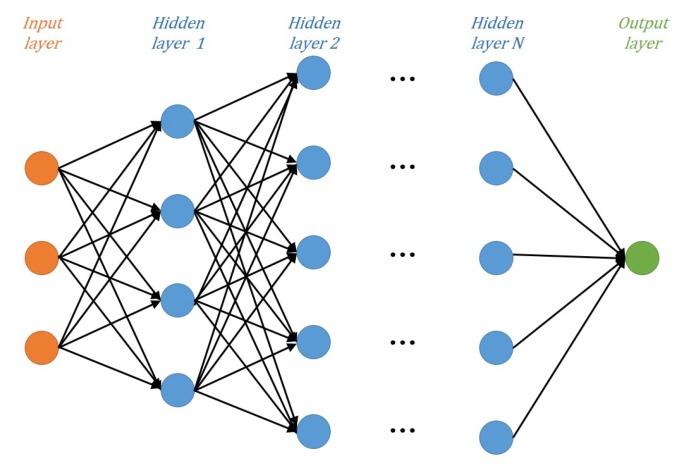
Construction of the deep neural network (DNN) model.

The DNN is a deeper version of the neural network that generally consists of three types of layers: input, hidden, and output. The input layer and output layer are single layers, whereas the hidden layers can be extended to multiple layers depending on the complexity of the signal-processing algorithm. Each layer contains multiple nodes, and the effects are exerted only on adjacent layers. The construction of DNN model can be seen in Figure 4.

There are two components of the relation between adjacent layers: linear and nonlinear. The linear component is responsible for the linear relation between the input and output for each layer. It includes two types of operation: multiplication, represented by the weight *w*, and addition, represented by the bias *b*. In most practical scenarios, however, we face nonlinear problems that cannot be solved by the linear method. Therefore, the nonlinear component is addressed via the activation function f(·).

Suppose that the output of the (n−1)th layer is $y_{n-1}$, the weight matrix of the *n*th layer is $w_{n}$, the bias vector is $b_{n}$, and the output of the *n*-th layer $y_{n}$ can be denoted as:

(6) $$y_{n}=f\left(w_{n}\cdot y_{n-1}+b_{n}\right).$$

A classic DNN activation function is the sigmoid function (7). The range of the function is limited to [0,1] and can approximately represent the probability. The tanh function (8) is also a classic activation function. The range of the tanh function is extended to [−1,1], and the center of each layer’s output is 0, which results in faster convergence via stochastic gradient descent (SGD). Another powerful activation function is the rectified linear unit (ReLU) function (9). Instead of restricting the value to [0,1] or [−1,1], the ReLU function increases linearly when x≥0 and is zero when x<0. The gradient does not disappear after repeated nonlinear operations.

(7) $$f_{sig}\left(x\right)=\frac{1}{1+\exp^{-x}}$$

(8) $$tanh\left(x\right)=\frac{\exp x-\exp-x}{\exp x+\exp-x}$$

(9) $$ReLU\left(x\right)=\left\{\begin{array}{cc} x & ,x\geq 0\\ 0 & ,x<0 \end{array}\right.$$

For the multiple hidden layers, assuming that the bias is 0 for simplicity, the transmission expression can be defined as:

(10) $$y_{n}=f(w_{n}f\left(w_{n-1}f\cdots w_{2}f\left(w_{1}y_{0}\right)\right)$$

For the output layer, the most popular choices are the sigmoid function expressed in (7) and the softmax function. The softmax function is used mainly for multiclass classification and can be defined as:

(11) $$f_{soft}{\left(x\right)}_{i}=\frac{\exp\left(x_{i}\right)}{\sum_{j}\exp\left(x_{j}\right)}.$$

In deep learning algorithms, we often need to feed the system considerable data, called the training set, so that the system can adjust itself adaptively to the optimal status offline. During the training process, correct data should be used to rectify the output. Then, a connection between the input and the output can be established in a supervised manner. Afterwards, the trained system can be applied to the test set to assess the performance of the DNN.

**Figure 5:**
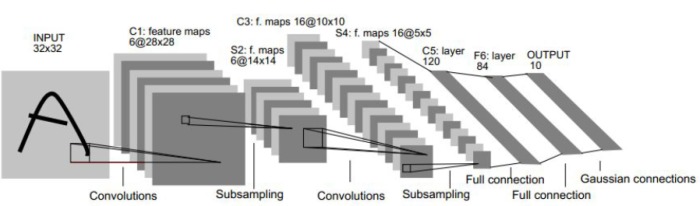
Construction of the LeNet-5.

In addition, CNNs also play an important role in DL, especially in the realm of computer vision. In contrast to the traditional NNs, CNNs have a special structure. A classic CNN is LeNet-5, which can be seen in Figure 5. Layer C1 is a convolution layer, S2 is a pooling layer, C3 is another convolution layer, and next is a pooling layer again in S4. C5 can be identified as a dense layer together with the F6 layer.

Obviously, before the fully connected layers, the input data are processed by multiple convolution layers and pooling layers. The convolution layer, which is composed of multiple feature maps, can dramatically reduce the number of connections via the convolution operation. The pooling layers, also called down-sample layers, can further compress the local data and avoid the overfitting problem. Max-pooling and mean-pooling are the most common choices to extract the maximum and mean values, respectively, from the output of convolution data according to the pooling size.

**Figure 6:**
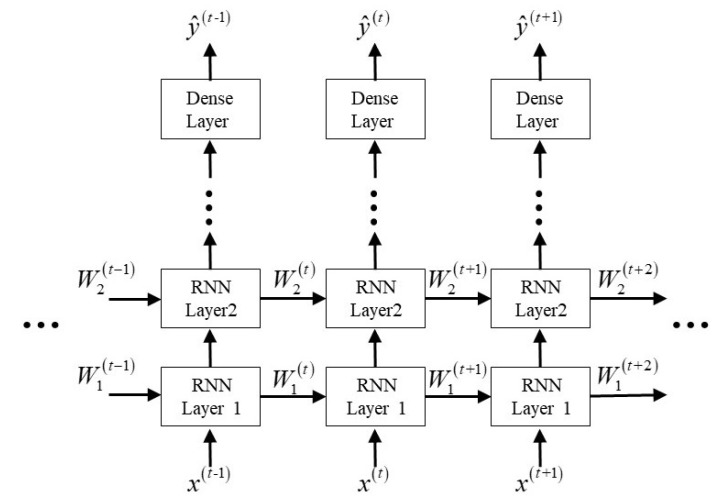
Construction of the recurrent neural network (RNN).

The RNN is another research hotspot in the field of NLP for its ability to memory the data. By establishing the relationship between current data and past data (and even future data), RNNs can deal with the situation where sequences at different slots have relations to each other. The basic structure of an RNN is shown in Figure 6. It can be seen that the information of previous data are summarized as state $W_{k}^{\left(t\right)}$ to solve the output ${\hat{y}}^{\left(t\right)}$ with the current input $x^{\left(t\right)}$. The number of RNNs’ output can differ from the input for various purposes, such as one input to many outputs for music generation, many inputs to one output for sentiment classification, and many inputs to many outputs for machine translation.

## 3. Deep-Learning Scheme Based on the MIMO-NOMA System

In wireless communication, signal detection can be considered to be a classification process of recovering a discrete sequence from an impaired signal. The DL technique has good ability to address this problem. Therefore, in this section, we consider a novel detector that adopts a DNN in a MIMO-NOMA system. In contrast to the traditional SIC block, which divides the process of detection into separate blocks, including channel estimation, MMSE detection, demodulation, channel decoding and signal decision, the deep learning method can perform all these procedures as a single process. The optimal parameters can be acquired by continuous iteration to determine the rules relating the output and the label.

### 3.1. Feasibility Analysis of DL in a MIMO-NOMA System

First, it should be proved that it is realizable to use DL to detect the MIMO-NOMA signal instead of an SIC receiver. We assume that the number of transmitting and receiving antennas is *M* and *N*. The number of UEs is *K*. The MIMO-NOMA transmission signal in (1) can be expressed in matrix form as (12):

(12) $$S=\left(S_{1},S_{2},\cdots ,S_{M}\right).$$

$S_{m}\left(m\in \left[1,M\right]\right)$ is the *m*-th transmission antenna and it can be expressed as:

(13) $$S_{m}=\sum_{k=1}^{K}\sqrt{P_{k}}S_{m}^{k}.$$

For M-ary phase shift keying (MPSK) modulation, $S_{m}^{k}\in X_{i}$ is the kth UE transmission signal of mth antenna, and $X_{i}$ is the set of *M* transmission signals:

(14) $$X_{i}=A\exp\left(j\omega t+\varphi_{i}\right)\;\left(i=1,2,\cdots ,M\right).$$

The MIMO-NOMA signal uses the power dimension to improve the channel capacity, so the channel matrix can be doted by a three order tensor $H\in R^{N\times M\times K}$. $h_{nm}^{k}$(k∈[1,K],m∈[1,M] and n∈[1,N]) is the channel gain of the *k*th UE from the *m*th transmitting antenna to the *n*-th receiving antenna.


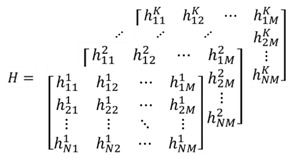
(15)

Transforming the tensor H into a matrix $H_{\left(n\right)}$ is called the node-*n* matricization [19]. Here, a mode-3 matricization $H_{\left(3\right)}$ can be expressed as:

(16) $$H_{\left(3\right)}=\left[\begin{array}{ccccccc} h_{11}^{1} & \cdots & h_{1N}^{1} & \cdots & h_{11}^{K} & \cdots & h_{1N}^{K}\\ h_{21}^{1} & \cdots & h_{2N}^{1} & \cdots & h_{21}^{K} & \cdots & h_{2N}^{K}\\ \vdots & \ddots & \vdots & \vdots & \vdots & \ddots & \vdots \\ h_{M1}^{1} & \cdots & h_{MN}^{1} & \cdots & h_{M1}^{K} & \cdots & h_{MN}^{K} \end{array}\right].$$

The mode-3 matricization of the received signal can be expressed as:

(17) $$Y_{\left(3\right)}=SH_{\left(3\right)}+N_{\left(3\right)}.$$

The channel gain and receiver signal of the *k*th UE are denoted by $H^{k}$ and $Y^{k}$, respectively:

(18) $$H^{k}=\left[\begin{array}{cccc} h_{11}^{k} & h_{12}^{k} & \cdots & h_{1N}^{k}\\ h_{21}^{1} & h_{22}^{k} & \cdots & h_{2N}^{1}\\ \vdots & \vdots & \ddots & \vdots \\ h_{M1}^{k} & h_{M2}^{k} & \cdots & h_{MN}^{k} \end{array}\right],$$

(19) $$Y^{k}=\left(\begin{array}{cccc} y_{1}^{k} & y_{2}^{k} & \cdots & y_{M}^{k} \end{array}\right).$$

The SIC detection needs a continuous process of decoding, reconstructing, and signal canceling. Assume that the power allocated to the UEs decreases gradually $\left(P_{1}>P_{2}>\cdots >P_{K}\right)$. $l_{q}^{k}$ is the estimation output of the *q*th UE signal at the *k*-th UE receiver. The channel state information (CSI) is perfectly known. For UE1, the MMSE detection process can be expressed as:

(20) $$l_{1}^{1}=\underset{i}{\arg\min}\;∥Y^{1}{\tilde{H}}^{1}-\sqrt{P_{1}}X_{i}∥,$$

(21) $${\tilde{H}}^{1}={\left(H^{1}\right)}^{H}{\left(H^{1}{\left(H^{1}\right)}^{H}+\sigma_{1}^{2}I\right)}^{-1}.$$

For UE2, the information of UE1 should be extracted first, and the process is similar to (20):

(22) $$l_{1}^{2}=\underset{i}{\arg\min}\;∥Y^{2}{\tilde{H}}^{2}-\sqrt{P_{1}}X_{i}∥,$$

(23) $${\tilde{H}}^{2}={\left(H^{2}\right)}^{H}{\left(H^{2}{\left(H^{2}\right)}^{H}+\sigma_{2}^{2}I\right)}^{-1}.$$

A reconstructed signal can be denoted as:

(24) $$S_{l_{1}^{2}}=A\exp\left(j\omega t+\varphi_{l_{1}^{2}}\right).$$

Then, the reconstructed signal is subtracted from the received signal:

(25) $${\hat{Y}}^{1}=Y^{2},$$

(26) $${\hat{Y}}^{2}={\hat{Y}}^{1}-\sqrt{P_{1}}S_{l_{1}^{2}}H^{2}.$$

Decoding the UE2 signal:

(27) $$l_{2}^{2}=\underset{i}{\arg\min}\;∥{\hat{Y}}^{2}{\tilde{H}}^{2}-\sqrt{P_{2}}X_{i}∥.$$

As the process mentioned above, for the *K*-th UE, the detection can be shown as:

(28) $${\hat{Y}}^{K}={\hat{Y}}^{K-1}-\sqrt{P_{K-1}}S_{l_{K-1}^{K}}H^{K},$$

(29) $$l_{K}^{K}=\underset{i}{\arg\min}\;∥\left({\hat{Y}}^{K}\right){\tilde{H}}^{K}-\sqrt{P_{2}}X_{i}∥.$$

From Equations (20), (27), (29), it can been seen that the final classification result can be expressed in the form:

(30) $$f_{K}\cdots f_{2}\left(Y_{2}f_{1}\left(Y_{1}A+b_{1}\right)+b_{2}\right)\cdots +b_{K},$$

where $Y_{k}$ is a constant matrix, $b_{k}$ is a constant vector, and $f_{i}$ represents some form of nonlinear function.

Comparing with (10), it can be found that the DNN detector is capable of replacing the traditional SIC method in a MIMO-NOMA system and is even more powerful due to its ability to find the optimal solution with the data-driven mode.

### 3.2. MIMO-NOMA-DL System

In this subsection, we propose a novel DL detector for MIMO-NOMA signal detection. Without any extra signal processing, the signal from the receiving antennas can be sent directly to the MIMO-NOMA-DL detector. Therefore, MIMO-NOMA-DL is an easier and more efficient scheme to replace the SIC receiver.

**Figure 7:**
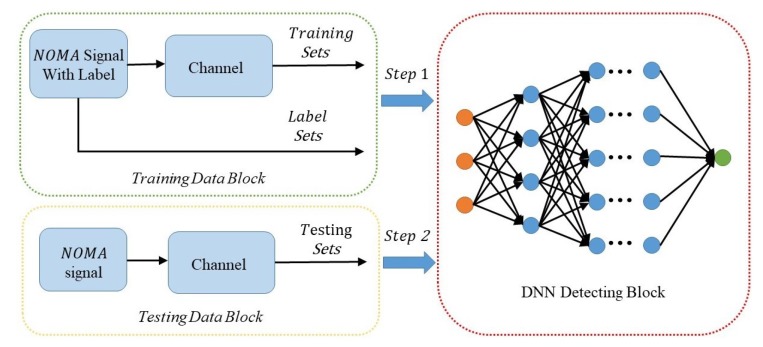
Construction of the MIMO-NOMA-DL model. DL: deep learning.

Overall, the MIMO-NOMA-DL system is composed of three components: training block, testing block, and DNN detecting block. The construction of the MIMO-NOMA-DL model is illustrated in Figure 7.

The training block is responsible for producing the MIMO-NOMA signal and providing the labels to the DNN. In this block, to acquire the MIMO-NOMA signal for $N_{t}$ antennas, we should produce two training sequences of UE1 and UE2 for each antenna. They are then modulated by superposition coding with different allocation power factors. After impairment by the fading channel and AWGN channel, the receive signal is acquired at the receiver. Meanwhile, these sequences are known by the receiver as labels, and it is similar to the pilot sequence.

The testing block is used to simulate real-time MIMO-NOMA transmission. In this block, we first produce the MIMO-NOMA signal. Labels are not required in this part. The testing data are used to assess the performance of the DNN detection. Notably, in order to avoid a perfect match, the channel models and the generated data in training blocks and testing block are i.i.d. to ensure that the DNN performs well in both the training and testing process. The SNR in training block is generated randomly with the data time slot varying over the range of interest, whereas the SNR in the testing block is fixed so that the error performance of the DNN can be evaluated in certain SNR conditions.

The DNN block is the main detection block for decoding the received signal. The channel characteristics and MIMO-NOMA decoding algorithm can be studied by optimizing the hyperparameters of the deep neural network. In this block, multiple parameters, including the number of layers, activation function, loss function, and optimization criteria iteration algorithm, must be designed; the details are discussed in the next subsection.

The first two blocks provide labels and signals polluted by channel, and the last block recovers the original data. Accordingly, the detection process can be divided into two steps:

- *Step 1: Training mode*
  In training mode, the offline training block is active while the online deployment block remains inactive. The input of the DNN training system includes two components: the received MIMO-NOMA signal as the input layer of the DNN system, and the labels as supervised data to help the DNN to optimize the parameters.
- *Step 2: Testing mode*
  The testing mode is activated after the DNN has been trained. In Step 2, the offline block is suspended, and the online block accesses the DNN system. The system performance is evaluated in this step, and the results of the simulation are presented in Section 4.

### 3.3. DNN Design

As mentioned above, a DNN is a deep version of a neural network. The adjustable hyperparameters include the weights, bias, regularization parameter, learning rate, and drop-out. The DNN model we designed for MIMO-NOMA detection comprises seven layers: one input layer, one output layer, and five hidden layers. The input layer and hidden layer are fully connected, whereas the output layers are divided into groups to decode the signals of multiple antennas in a slot.

The input layer is where the MIMO-NOMA signal is received. Suppose that the numbers of transmit and receive antennas of the BS and UE are $N_{t}$ and $N_{r}$, respectively. The complex receive signal is decomposed into the real part and the imaginary part, so the number of input layer cells is $2N_{r}$. The input signal is a two-dimensional vector of a slot and multiple antennas. That is, the $2N_{r}$ data are sent to the network in one slot as a column vector.

The hidden layers are composed of five fully connected layers. To avoid the vanishing gradient problem of the sigmoid function, the ReLU function (9), an effective nonlinear function, is used to activate the neurons after the linear operation.

**Figure 8:**
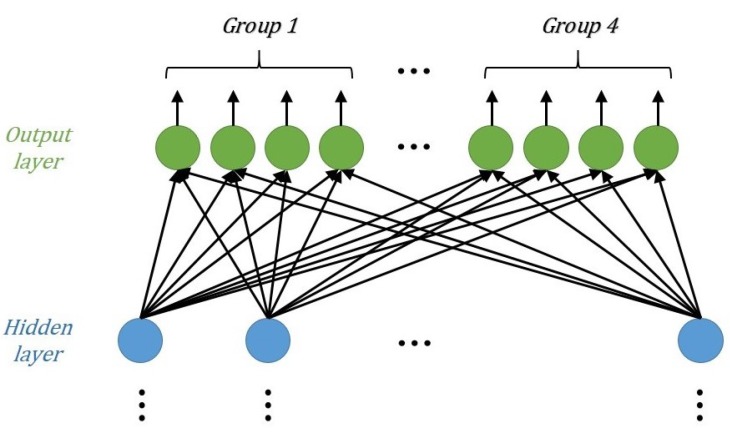
Design of the output layer.

The output layer is used to report the final detection results. The normal DNN output layer is usually fully connected, and has one-hot encoding with the softmax function. In MIMO-NOMA signal detection, however, signals from multiple antennas should be decoded in a single slot. So, the proposed output layer was designed to form groups. The number of groups is equal to the number of transmitting antennas $N_{t}$, and the number of neurons in each group is equal to the number of one-hot encodings. For example, the output layer structure of a 4×4 MIMO-NOMA system is shown in Figure 8. Because the label is not in the traditional form, but the group one-hot encoding, the output data adopt the soft-decision form with the sigmoid function (7).

Additionally, the choice of loss function and optimization algorithm is another key point for the MIMO-NOMA-DL network. The loss function measures the distance between the predictions and labels. The classic loss function is the mean square error (MSE) function. In logistic regression, MSE performs in terms of accuracy. However, in multiclass classification problems, MSE has a slow convergence speed. Here, we consider the cross-entropy function. In Shannon’s information theory, the Kullback–Leibler divergence (KLD) can be used to represent the difference between two probability distributions, and the expression is written as:

(31) $$D_{KL}\left(P||Q\right)=\sum_{i}P\left(i\right)\log\frac{P(i)}{Q(i)}\left(x\right).$$

The process of minimizing the KLD is equivalent to minimizing the cross-entropy H(P,Q), which is defined as:

(32) $$H\left(P,Q\right)=D_{KL}\left(P||Q\right)+H\left(P\right).$$

The cross-entropy function has fast convergence and low complexity in the iterative optimization process.

Moreover, considering the self-adaptation of the learning rate and the robustness, the Adam [20] method is used as the optimization algorithm. The Adam method improves upon the Momentum and RMSPro algorithms, and is more robust in terms of the hyperparameters. Details of the optimization algorithms and their comparison can be found in [21].

To avoid overfitting, we add the L2 regularization term $\lambda \cdot \sum_{i}∥\omega_{i}{∥}_{2}$ to the loss function to effectively decrease the sensitivity of the parameters to parametric variation so that the performance of the test result is close to the training result.

The proposed DL algorithm based on the above DNN architecture design is summarized in Algorithm 1.

## 4. Simulation and Analyses

In this section, we build the NOMA signal detection system based on the deep learning method and present the numerical results for different parameters. First, we investigated the performance of the proposed scheme in comparison with the traditional SIC method in a certain scenario. Then, the influence of diverse types of MIMO-NOMA modulations on the symbol error rate (SER) performance was studied. Next, we simulated the impact of the power allocation factor on the system performance. Then, we explored the situation where the estimated CSI is deviated from the actual one. Additionally, to achieve faster convergence of the MIMO-NOMA-DL algorithm, we conducted simulations with different mini-batch sizes. Finally, useful recommendations to accelerate training are provided.

Many software and tools are available for machine learning. Considering their efficiency and usability, two of the most popular tools—Python 3.6 and MATLAB—were used in our numerical analysis. As a powerful open-source machine-learning framework from Google, TensorFlow with GPU acceleration was also employed to implement the proposed deep learning algorithm. For simplicity, we concentrated on a single cluster with two UEs. A 4×4 MIMO channel with a complex Rayleigh distribution was considered. The total transmitted power for one antenna was set to 1 W. UE1 was allocated 80% of the power, and UE2, 20%. The activation function of the output layer was the sigmoid function (7) and that of the hidden layers was the ReLU function (9). The total number of training samples was 409,600, in the form of the *n*-power of 2, so we could use a smaller data set—mini-batch—to accelerate the convergence. For the 4×4 NOMA-MIMO signal, the number of input layer cells in a slot was 8, and the input data were fed to the DNN as a column vector. All the labels used in the supervised training were one-hot encoded. The key parameters are summarized in Table 1, and the detailed algorithm is depicted in Algorithm 1.

**Algorithm 1** MIMO-NOMA Based on the DL Training Algorithm.
1:Initialize the DNN model;2:Generate and adjust the format of the training data. Assuming that the number of slots is *N*, the input data are denoted as $x=\{x^{\left[1\right]},x^{\left[2\right]},\cdots ,x^{\left[N\right]}\}$. Each $x^{\left[i\right]}$ is a MIMO-NOMA column vector in slot *i*;3:Set the key parameters, including mini-batch, learning rate, and output functions of the hidden layer and output layer, and initialize the weight and bias of each DNN layer;4:Implement the forward DNN process and obtain the results of the output layer’s data, denoted as ${\hat{y}}_{i}=\left\{{\hat{y}}_{i}^{\left[1\right]},{\hat{y}}_{i}^{\left[2\right]},\cdots ,{\hat{y}}_{i}^{\left[N\right]}\right\}$;5:Calculate the loss function, that is, the cross-entropy $Loss(y,\hat{y})$
(33)$Loss\left(y,\hat{y}\right)=\sum_{i}y_{i}\log\frac{y_{i}}{{\hat{y}}_{i}}+H\left(y\right)+\lambda \cdot \sum_{i}∥\omega_{i}{∥}_{2}.$6:Calculate the corrective parameter with the Adam optimization algorithm. Update the parameters with the algorithm to search for the optimal solution;7:Return to Step 4 if the loss function is not small enough, otherwise proceed to the next step. If the loss function does not meet the requirement, the DNN with the updated parameters should be retrained;8:Test the trained DNN with the test data and plot the SER–SNR curve.


**Figure 9:**
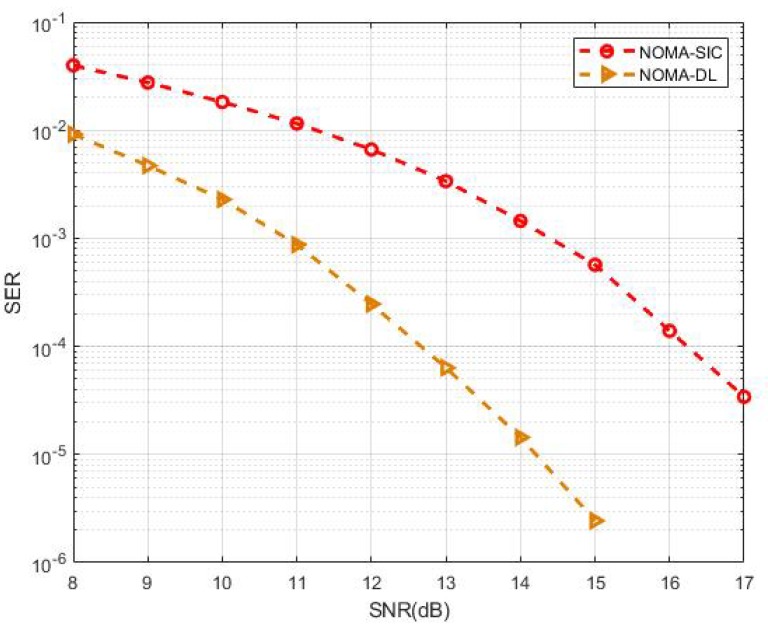
Performance comparison of MIMO-NOMA-DL and MIMO-NOMA-SIC.

To evaluate the performance gap, the proposed MIMO-NOMA-DL signal detection was compared with the traditional MIMO-NOMA-SIC scheme. We assumed that the SIC had perfect knowledge of channel parameters and that the modulation type of both UE superposition coding signals at the transmitter was binary phase shift keying (BPSK). In the traditional MIMO-NOMA-SIC detection scheme, the UE1 signal—which treats the UE2 signal as interference—should be demodulated first. Then, we can demodulate the UE2 signal after the modulated UE1 signal is removed from the received NOMA-MIMO signal. In the MIMO-NOMA-DL scheme, however, the received signal is sent to the DNN, and labels are chosen only for the UE2 sequence during the training step. Figure 9 shows the SER–SNR curve of the numerical simulation. Taking $10^{-4}$ as the standard to measure performance gain, we can see that the proposed MIMO-NOMA-DL reached 12.6 dB, whereas the traditional scheme reached 16.2 dB—a difference of approximately 3.6 dB. Notably, no preprocessing nor postprocessing was performed. Instead of the traditional complex signal processing for channel estimation and signal demodulation, we used powerful deep learning tools to perform accurate signal detection. That is, we used a computer to automatically search for the most rational schemes for channel estimation and signal demodulation to avoid complicated human designs, which is the main reason we achieved performance gains.

As indicated previously, the MIMO-NOMA system adopts superposition coding in which signals from different UEs in a cluster are overlaid with the specific proportion of the power. Different modulation types can be used for diverse UE signals. Because power-field NOMA is a type of non-orthogonal technology, interference from other UEs—especially the type of their modulation—is a significant factor in determining the demodulation performance. Here, we simulated several groups of different types of modulation from the most common PSK modulation, and we assessed whether the NOMA-DL system had good performance in these situations. Table 2 shows three groups of simulation parameter settings, including the situation where both UEs had BPSK or quadrature phase shift keying (QPSK) modulation and where one UE used BPSK modulation while the other used QPSK. Considering that decoding the UE2 signal requires the UE1 signal to be decoded first according to the SIC method, we considered only the UE2 signal detection in the DL method due to its higher complexity.

**Figure 10:**
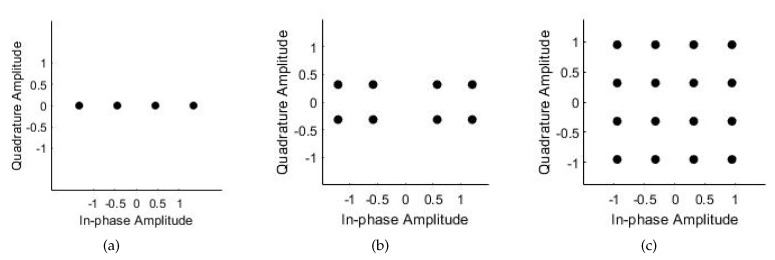
Constellation of UE signal superposition coding: (**a**) BPSK and BPSK, (**b**) BPSK and QPSK, and (**c**) QPSK and QPSK.

Figure 10 shows the modulated MIMO-NOMA signal constellation graph for all three cases. The initial phases of BPSK and QPSK were 0${}^{\circ }$ and 45${}^{\circ }$, respectively, and there were two UEs in a cluster. The allocation of the transmit signal power affects the Euclidean distance between constellation points and the error probability. Here, we were not concerned with the power distribution, and set the power allocation factor to 0.8 for simplicity.

**Figure 11:**
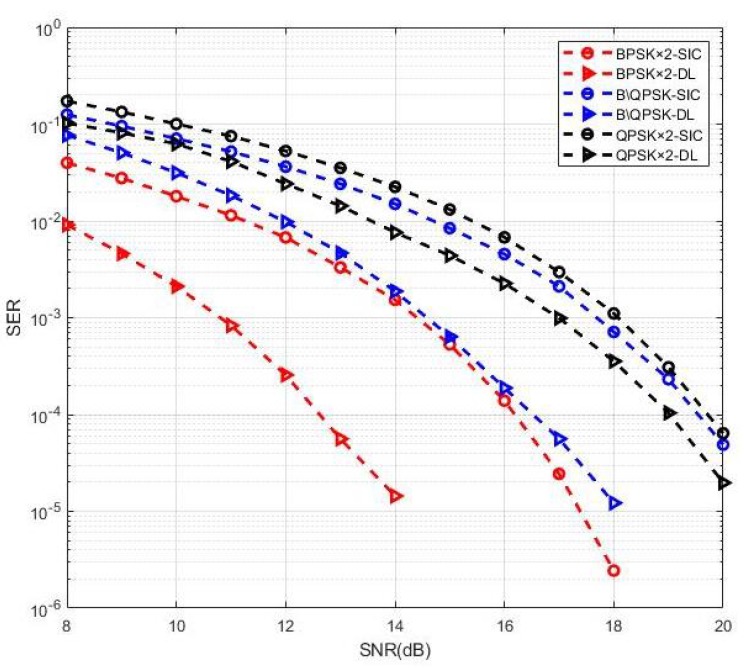
Performance comparison of MIMO-NOMA-DL and MIMO-NOMA-SIC.

Figure 11 depicts the detection performance in these three cases. Clearly, the MIMO-NOMA signal detection based on the DL system had good performance. Besides case 1 mentioned above, nearly 3.5 dB performance gain was achieved in case 2, and the gain was 1 dB in case 3. These results indicate that both the characteristics of the wireless MIMO channel with Rayleigh fading and the signal demodulation with NOMA could be learned through the DNN.

**Figure 12:**
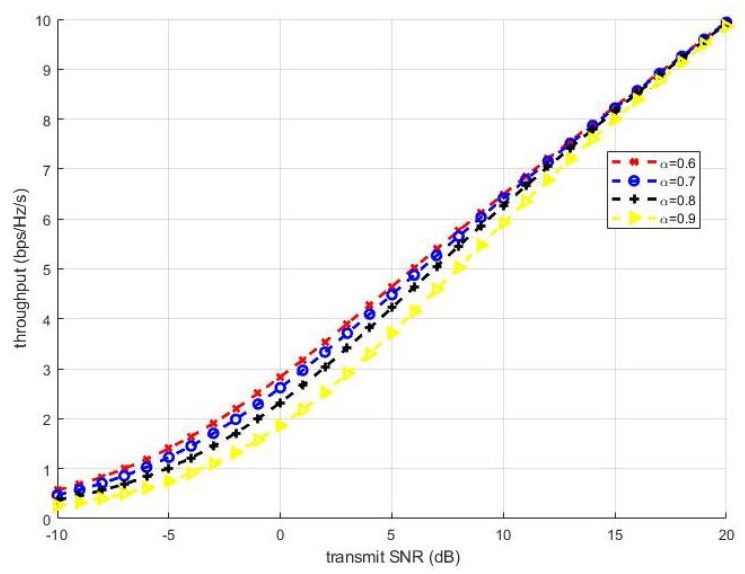
NOMA throughput with different α values.

The allocation of the transmit power of the UEs at the BS is a crucial component of the conventional NOMA scheme, and has a substantial impact on the NOMA throughput with Shannon’s information theory. Several studies have provided optimal or suboptimal allocation methods. According to (5), the sum throughput of two UEs can be denoted as:

(34) $$R_{sum}\left(\alpha \right)=\log_{2}\frac{\left(1+\left(1-\alpha \right)\rho {\left|h_{2}\right|}^{2}\right)\left(1+\rho {\left|h_{1}\right|}^{2}\right)}{1+\left(1-\alpha \right)\rho {\left|h_{1}\right|}^{2}},$$

where ρ is the transmit SNR. $h_{i}\left(i=1,2\right)$ is the channel gain, $h_{1}<h_{2}$. The throughput can be shown to be a monotonically decreasing function of α for $\frac{dR_{s}um}{d\alpha }<0$, where α∈[0,1]. Figure 12 shows the throughput of the NOMA system with power allocation factor fixed at 0.6, 0.7, 0.8, and 0.9. The throughput increased as the transmit SNR increased. Moreover, the larger the power allocation factor was, the smaller the throughput.

**Figure 13:**
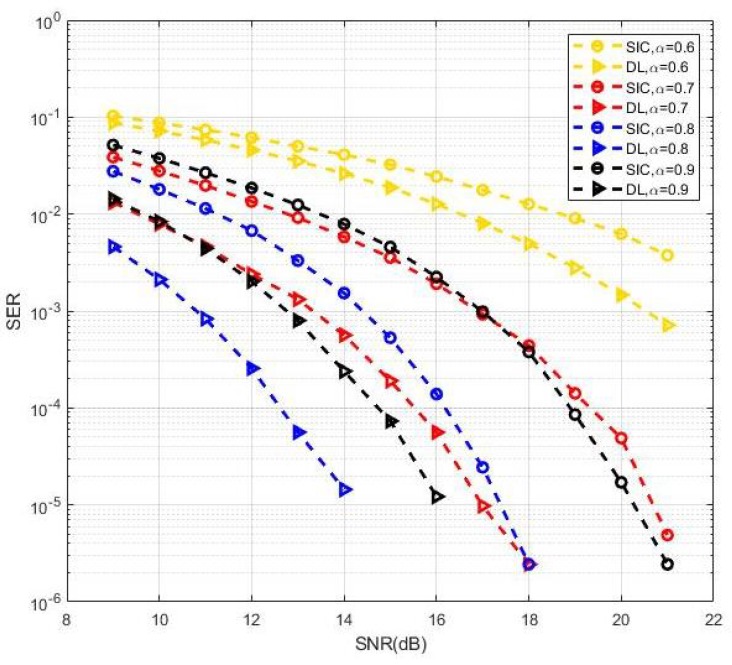
SER performance with different power allocation factors.

Usually, the throughput of different UEs should satisfy the quality of service (QoS) requirements, or the optimal power should be allocated according to the feedback of the channel state. Here, we considered the performance of the proposed methods from the perspective of reliability. Figure 13 shows the SER performance in MIMO-NOMA signal detection for different power allocation factors with BPSK modulation. The proposed DL-based methods achieved good SER performance compared with the SIC receiver in all cases of power allocation factors. Furthermore, a power allocation factor of 0.8 appeared to be closest to the optimal solution in terms of the minimum symbol error probability. Here, we considered some specific fixed power allocation factors on MIMO-NOMA signal detection. The optimal power allocation factors to minimize the SER performance and sum throughput for DL methods in cases of multiple users and clusters can be considered in our follow-up study.

**Figure 14:**
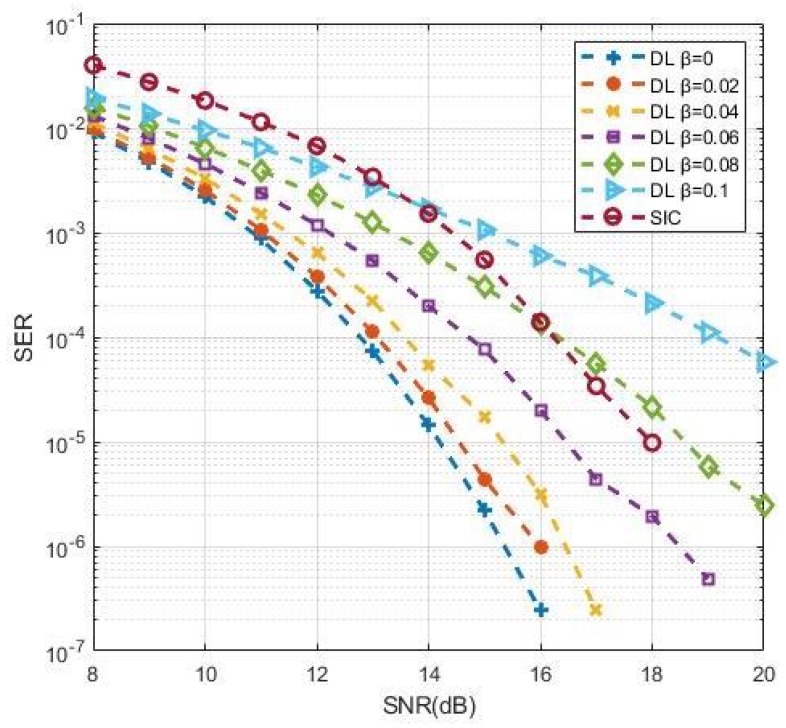
SER performance with channel estimation error.

The process of channel estimation and detection was finished at the training stage. By introducing the error into the channel at the testing stage, we explored how the proposed DL approach behaved when the estimated CSI deviated from the actual situation. The channel error model can be denoted as:

(35) $$H=\hat{H}+\beta \Omega ,$$

where H is the actual channel matrix impaired by error. $\hat{H}$ and Ω are the original Rayleigh channel matrix and the channel error matrix, respectively, and they are i.i.d. β is the error factor. Figure 14 shows the impact of channel estimation error on the DL-based approach. It can be seen that the SER deteriorated gradually with the increase of β from 0 to 0.1. Compared with the SIC method with perfect CSI, the DL approach still showed superiority when β was less than 0.08. This result explains that the performance suffered losses when deviations appeared between the estimated and actual CSI, though the performance of the DL-based approach could keep its predominance within a specified tolerance range.

**Figure 15:**
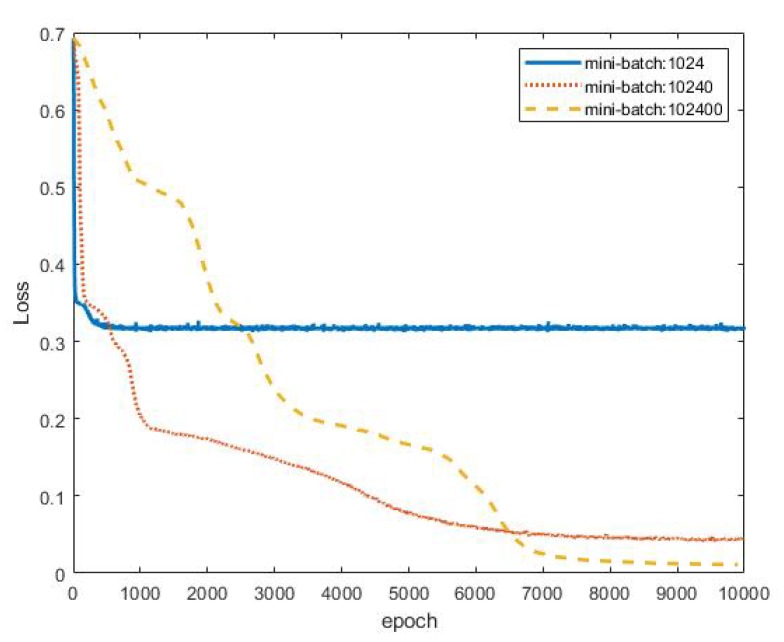
Loss function for different mini-batch sizes in the MIMO-NOMA-DL method.

Restricted by the performance of the CPU/GPU/RAM, the convergence of deep learning algorithms is often time consuming when addressing a large amount of data. Mini-batch gradient descent is a good way to shorten the training run-time to obtain the results efficiently. Here, different mini-batch sizes in the MIMO-NOMA-DL detection method were studied to provide a reference for related research. Figure 15 shows the loss values for three mini-batch sizes. The loss value for a mini-batch size of 1024 reached 0.32 at approximately 360 epochs. A mini-batch size of 1024 resulted in the fastest convergence but the worst loss value. By contrast, a mini-batch size of 102,400 produced the best loss performance but the longest run-time: the loss value tended towards stability at 0.015 at approximately 8000 epochs. The middle mini-batch size of 10,240 achieved moderate performance. After 6000 epochs, the loss value approached 0.55. Hence, the choice of mini-batch size is a trade-off between convergence speed and error precision. The most suitable mini-batch size can be identified to meet the specific requirements. In this article, we recommend that a smaller mini-batch size be used first. When the loss tends toward stability, the mini-batch size can be increased until the loss value reaches the required precision.

As mentioned above, the computational complexity of SIC is related to the number of UEs. Suppose the number of UEs in a cluster is *L* and the computational complexity of SIC is O(L). For a trained DNN model, the signal detection is the process of forward propagation, and its computational complexity is O(1). This means that the trained DNN model can realize efficient and real-time signal detection.

## 5. Conclusions

The application of deep learning in MIMO-NOMA communication systems is a promising approach to address the shortcomings of the SIC method. Instead of the complicated algorithm design and interference cancellation process, the deep learning approach can search for the optimal solution of the hyperparameters of the multilayer neural network with machine learning.

In this paper, we designed an MIMO-NOMA-DL signal-detection system to perform signal recovery. The proposed technique can simultaneously complete the processes of channel estimation and MIMO-NOMA signal detection. The detailed construction and learning algorithm have been provided. We first compared the SER performance of the proposed method and the SIC algorithm via simulations. The highest performance gain reached 3.6 dB. Then, the impact of the crucial parameters, including the modulation type and power allocation, were studied. Numerical results showed that the MIMO-NOMA-DL method had powerful detection performance. Finally, mini-batch gradient descent simulations were conducted to accelerate the training step of the MIMO-NOMA-DL algorithm. The results indicate that the mini-batch size is a key parameter for balancing the convergence speed and loss precision.

Future works will explore the DL-based approach to detect other types of NOMA signals, such as the sparse code multiple access (SCMA), multi-user shared access (MUSA), and pattern-division multiple access (PDMA). Moreover, we also consider an extension assessing the performance under different channel situations and the multiple clusters situation. Additionally, detecting the communication signal with memory using RNNs will be explored. CNNs, another advanced DL approach, could be deeply developed in terms of their potential in signal detection as our following work.

## Figures and Tables

**Table 1:** Parameter settings used in the simulation. ReLU: rectified linear unit.

Parameter	Value
Operating system	Windows 7
Framework	TensorFlow
Programming language	Python 3.6/MATLAB
Channel	MIMO channel/AWGN
Fading	Rayleigh distribution
Number of UEs per cluster	2
Number of transmit antennas	4
Number of receive antennas	4
Modulation	PSK
Number of training samples	409,600
Total transmitted power per antenna	1 W
Power allocation factor	0.8
Hidden layer	ReLU
Output later	Sigmoid

**Table 2:** Parameter settings used in the simulation.

Modulation Scheme	UE1	UE2
1	BPSK	BPSK
2	BPSK	QPSK
3	QPSK	QPSK
